# Diet-induced deficits in goal-directed control are rescued by agonism of group II metabotropic glutamate receptors in the dorsomedial striatum

**DOI:** 10.1038/s41398-022-01807-2

**Published:** 2022-01-28

**Authors:** Megan L. Shipman, Laura H. Corbit

**Affiliations:** University of Toronto Department of Psychology, 100 St. George Street, Toronto, ON M5S 3G3 Canada

**Keywords:** Learning and memory, Psychology

## Abstract

Many overweight or obese people struggle to sustain the behavioural changes necessary to achieve and maintain weight loss. In rodents, obesogenic diet can disrupt goal-directed control of responding for food reinforcers, which may indicate that diet can disrupt brain regions associated with behavioural control. We investigated a potential glutamatergic mechanism to return goal-directed control to rats who had been given an obesogenic diet prior to operant training. We found that an obesogenic diet reduced goal-directed control and that systemic injection of LY379268, a Group II metabotropic glutamate receptor (mGluR2/3) agonist, returned goal-directed responding in these rats. Further, we found that direct infusion of LY379268 into the dorsomedial striatum, a region associated with goal-directed control, also restored goal-directed responding in the obesogenic-diet group. This indicates that one mechanism through which obesogenic diet disrupts goal-directed control is glutamatergic, and infusion of a mGluR2/3 agonist into the DMS is sufficient to ameliorate deficits in goal-directed control.

Obesity, and associated disease burden, is a preventable global health problem with over half of the adult population being classified as overweight or obese in 2016, equating to 1.9 billion people worldwide [1]. Despite being associated with many negative health consequences and reported high motivation for weight loss, people frequently struggle to maintain weight-loss long term [2]. This may be because many eating behaviors are habitual and resistant to change. Indeed, a more effective way of maintaining weight loss may be by targeting habits that have developed in relation to food [3]. In animal research, long-term access to a highly palatable diet itself results in the loss of behavioural control as measured by sensitivity of behaviour to outcome devaluation [4–6]. This may indicate a problematic cyclical process where habits that result in higher consumption of unhealthy foods alter brain regions responsible for behavioural control, thus further promoting existing habits.

Normally, instrumental responding is initially sensitive to changes to the value of its earned outcome. However, across extended training, behaviour becomes habitual and relatively insensitive to changes to outcome value. Indeed, the hallmark of a habit is that behaviour, once established, continues without consideration of the outcome of responding, as stimuli associated with making a response for a reinforcer trigger responding independent of the reinforcer itself. Though this is generally adaptive, allowing well-practiced tasks with a strong history of reinforcement to be efficiently executed and freeing cognitive resources, certain experiences can prematurely shift control of responding to habitual. A history of an obesogenic diet is one such experience that can promote early habitual responding. Furlong et al. [4] showed that feeding rats a diet of sweetened condensed milk (SCM) for five weeks prior to instrumental training, where rats learned to press a lever for a different food reinforcer, resulted in insensitivity to the devaluation of that outcome following an amount of training where control subjects were sensitive to devaluation.

Diets high in sugar and fat (including so-called Western diets or cafeteria diets), and drugs of abuse (e.g., cocaine, amphetamine, methamphetamine, and alcohol) appear to have many overlapping neural and behavioural effects [7–11]. In particular, noncontingent administration of both drugs and palatable foods prior to instrumental training for food reinforcers promotes habitual responding [12–19]. This impact on future learning indicates that diet, like drugs, must induce plasticity in brain circuitry involved in behavioural control. This early insensitivity to devaluation could be due to glutamatergic adaptations in the striatum, as changes to glutamate receptor expression and basal glutamate transmission have been found to be altered following treatments that promote early habitual control [4, 12, 13].

In a related literature, group II metabotropic glutamate receptors (mGluR2/3) have been implicated in regulating drug and food self-administration and reinstatement [20]. Withdrawal from cocaine following chronic exposure is associated with a reduction in basal extracellular levels of glutamate in the nucleus accumbens, while a priming dose of cocaine increases extracellular glutamate [21]. Most mGluR2/3 receptors are presynaptic autoreceptors positioned extrasynaptically that serve to negatively regulate glutamate release at the synapse. They are typically stimulated by glutamatergic spillover from the synapse and by glutamate release from astrocytes, which maintains basal glutamatergic tone [22, 23]. Systemic injection of LY379268, a mGluR2/3 agonist, inhibits self-administration of cocaine, alcohol, methamphetamine, nicotine, and food [24–29] as well as reinstatement of responding for drugs of abuse [24, 26–28, 30, 31]. It also has a moderate effect on reinstatement of food-seeking [32, 33]. Drug-seeking and reinstatement have also been attenuated with LY379268 injection directly into the nucleus accumbens shell [27, 34, 35] or the ventral tegmental area [27].

Though there is much evidence that mGluR2/3 receptor stimulation can reduce drug-seeking and reinstatement, it is untested whether manipulation of mGluR2/3 receptors might affect habitual responding that also preserves drug use or overeating. N-acetylcysteine (NAC), a drug that drives cystine/glutamate transporters, thus increasing extrasynaptic glutamate, attenuates cocaine-induced reinstatement and this effect is blocked with antagonism of mGluR2/3 receptors [16, 31]. Systemic NAC can also reverse cocaine-induced deficits in goal-directed responding and return a cocaine-induced increase of EPSPs in the dorsomedial striatum to baseline [12]. Crucially, NAC-induced return of behavioural control demonstrates that it doesn’t simply inhibit reward-seeking but restores flexible responding. Since cocaine and an obesogenic diet produce similar behavioural deficits, and it has been shown that the cocaine-related impairment can be reversed by NAC treatment; if a similar mechanism underlies diet-induced deficits, more specific agonism of mGluR2/3 receptors should also improve goal-directed control.

Therefore, we tested the hypothesis that an obesogenic diet would impair goal-directed control in rats and that this would be ameliorated by mGluR2/3 agonism both systemically and by direct infusion into the dorsomedial striatum (DMS), a region known to be essential for goal-directed learning and performance [36, 37].

## Methods

All procedures were conducted in accordance with ethical standards set by the Animal Care and Use Committee at the University of Toronto and guidelines established by the Canadian Council on Animal Care.

### Experiment 1

#### Subjects

This experiment was run in two cohorts with all procedures matched as closely as possible apart from some details of training and an early test conducted in the first cohort which are explained in detail below. The first cohort consisted of 12 male and 12 female Long Evans rats, and the second cohort consisted of 16 male rats and 16 female rats (Charles River, St. Constant, QC, Canada). These numbers were chosen because similar work has shown that approximately 12 rats per group is adequately powered. All rats were approximately 8 weeks old at the time of arrival. Rats were pair-housed in individually ventilated cages with free access to chow and water and environmental enrichment (e.g., red tubes and nyla bones). Housing rooms were maintained on a 12:12 light/dark cycle with lights on at 7 am. Rats were allowed one week to acclimate before the diet manipulation began.

#### Apparatus

Training took place in operant chambers (Med Associates, East Fairfield, Vermont) housed within sound- and light-attenuating shells. The chambers were equipped with a pellet dispenser that delivered one 45 mg pellet when activated (grain-based formula; BioServ FO165) and a syringe pump that delivered 0.1 ml of a 20% sucrose solution when activated. The chambers contained a retractable lever that could be inserted to the left of the magazine. A 3 W, 24 V houselight mounted on the wall across from the lever and magazine illuminated the chamber. Computers equipped with MED-PC software (Med Associates) controlled the equipment and recorded responses.

#### Obesogenic diet

**Fig. 1:**
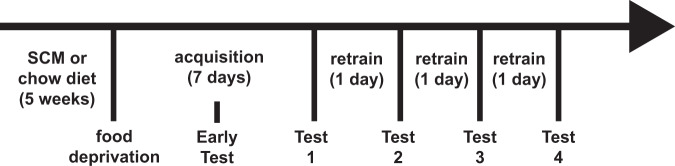
Timeline for Experiment 1. Rats received 5 weeks of SCM or diet followed by instrumental training and four tests to evaluate sensitivity to outcome devaluation under drug and control conditions.

Rats were matched on start weights and assigned to sweetened condensed milk (SCM) or control groups. SCM (Selection brand: 70 cal/1.5 g fat/1 g saturated fat/11 g sugar per 15 ml) was diluted with filtered water (3 parts SCM to 1 part water) and rats had continuous access to this solution as well as to water in a separate bottle while in the home cage. At approximately the same time each day, the weight of the remaining milk was recorded, and consumption calculated. Control animals had continuous access to chow and water. All rats were weighed daily. The diet phase continued for five weeks and ended prior to instrumental training (See Fig. 1 for a timeline).

#### Food deprivation

**Fig. 2:**
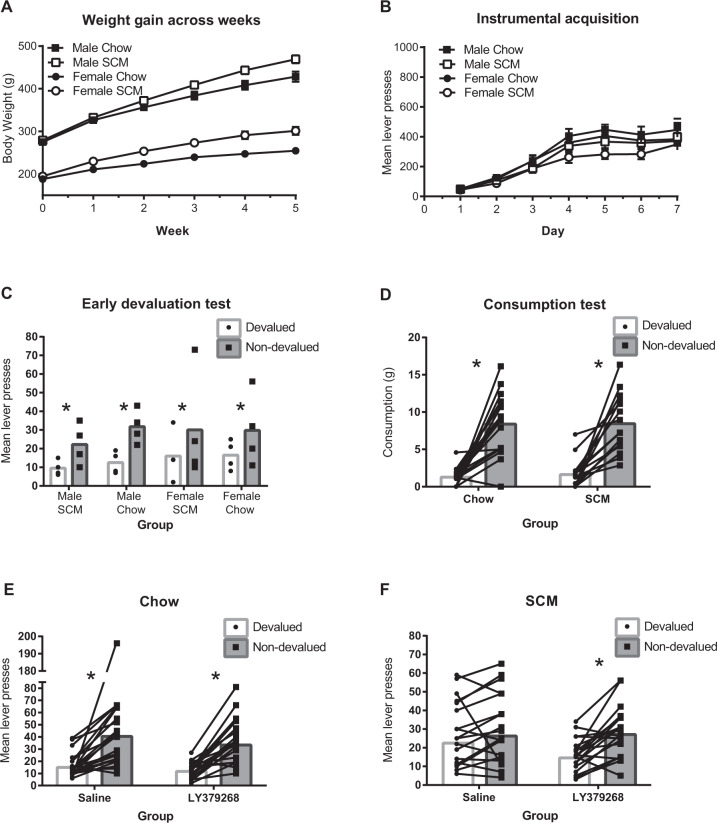
Long-term access to an obesogenic diet reduces sensitivity to outcome devaluation and sensitivity is restored by mGluR2/3 agonism. **A** Weight gain. Mean (SEM) weight gain across weeks of the diet treatment. All rats gained weight across five weeks of a SCM or Chow diet. Rats in the SCM groups gained more than those receiving only chow and males gained more than females. **B** Instrumental acquisition. Mean (SEM) lever presses across days of instrumental training. All animals increased lever-pressing across days of training. **C** Early devaluation test. An early outcome devaluation test showed no difference in sensitivity to devaluation between groups receiving Chow or SCM diets nor between males and females. **D** Consumption test of outcome devaluation. All animals showed sensitivity to outcome devaluation when measured as consumption of the prefed (devalued) versus alternative (nondevalued) food. **E** Outcome-devaluation test: Chow group. Rats maintained on a chow diet are sensitive to outcome devaluation. Responding overall was attenuated following administration of LY268379 (1 mg/kg) but sensitivity to devaluation was robust in both drug conditions. **F** Outcome-devaluation test: SCM group. A history of access to an obesogenic diet reduces sensitivity to outcome devaluation. This deficit was ameliorated by systemic mGluR2/3 agonism with LY379268 (1 mg/kg). N = 26 for the Chow and 27 for the SCM groups, respectively. Across all figures, * indicate that *p* < 0.05.

Rats in both groups were food restricted for 3 days prior to the start of magazine training. All animals were given the same ration of chow (15 g per male rat, 10 g per female rat, per day) aimed at maintaining controls at 90% of their free-feeding weight, although body weights for chow and SCM groups differed following the diet treatment and throughout training (see Fig. 2A).

#### Pre-exposure and habituation

To familiarize rats with the novel foods and facilitate acquisition and consumption in the devaluation tests, 20 mls of sucrose and 20 individual pellets were placed into the home cage to reduce neophobia before training. The rats were also habituated to the individual cages where the devaluation procedure would take place.

#### Magazine training

Earned outcome (pellet or sucrose solution) was counterbalanced within each group (SCM vs control) and with an attempt to match body weight following the diet treatment. Rats received 1 day of magazine training in which the reinforcer that would be earned in instrumental training was delivered on average once a minute during the 20-min session. No lever was available.

#### Operant training

The house light was illuminated, and the lever inserted at the beginning of the session. Rats received reinforcement on progressively lean variable interval (VI) schedules. In the initial cohort, each session stopped once 40 reinforcers were delivered or 60 min elapsed. Rats were required to earn at least 30 reinforcers to proceed to the next schedule. On the first day, lever-press training was continuously reinforced followed by one day of VI15, one day of VI30, and one day of VI60. Because of a recent report that female rats may form habits after minimal training [38] which could preclude us detecting the predicted effect of diet in female rats, in the first cohort of rats we conducted an initial drug-free devaluation test after the first VI60 training session at which point, rats had earned only 160 outcomes. As all rats showed robust devaluation in this test, we went on to conduct further training (6 days of VI60, with 40 outcomes earned per session) until animals had earned 400 outcomes, an amount of training more typical of our related work [4, 7]. Because we found no sex differences and no evidence of early habit formation in either sex in the first cohort, the second cohort was trained following the procedures of Furlong et al. [4]; following magazine training, lever pressing was continuously reinforced for 1 day, followed by one day of VI15, one day of VI30, and four days of VI60 prior to the first devaluation test. These sessions stopped when 60 reinforcers were delivered or 75 min had elapsed thus, rats had earned 420 outcomes prior to the first test.

#### Outcome-devaluation tests

An early test was conducted drug free in rats from the first cohort only. Rats in each diet condition were assigned to either devalued or nondevalued conditions in an attempt to match response rates from training. Rats had free access to either the previously earned outcome (devalued condition) or alternative food (nondevalued condition) for 1 h in a previously exposed individual cage after which they were transferred to the operant chamber for a 5-min extinction test where the lever was available, but no outcomes were delivered. After this test, rats in the first cohort received further training (see above) before being tested again as follows.

Rats within each diet condition were assigned to drug groups in a counter-balanced manner based on lever pressing rates during the last training session. Rats had free access to either the previously earned outcome (devalued) or the alternative food (nondevalued). Following an hour with free access to the food, animals were injected (IP) with either LY379268 (1.0 mg/kg) or saline (1.0 ml/kg). Following injections, rats were returned to their home cages for 30 min prior to being placed in their respective operant chambers. Tests occurred in extinction for 5 min where levers were available, but no outcomes were delivered. Following a retraining session (VI60), rats were retested under the same drug condition but were prefed the alternative food, thus reversing the devaluation condition. Animals were retrained again (VI60) before another pair of tests (devalued and nondevalued) that reversed drug treatments. Male rats who consumed less than 3 g of pellets or 10 ml of sucrose and female rats who consumed less than 2 g of pellets or 10 ml of sucrose during the 1 hr free access period were not tested that day but retested under the same condition the following day.

#### Consumption test

Rats were given a follow-up test in which they were each given one food in the same manner as the devaluation tests described previously. After 1 h of access to that food, it was removed, and fresh containers of both pellets and sucrose were placed into the cage for 20 min and consumption of each recorded. This was to determine the effectiveness of specific satiety and to verify that this was not altered by SCM exposure (See Fig. 2D).

#### Statistical Analysis

Data were analyzed using mixed ANOVAs. Interactions were further examined with further ANOVA or paired *t*-tests to isolate the contribution of specific factors.

### Experiment 2

#### Subjects

Sixty-four Long Evans rats (Charles River, St. Constant, QC, Canada) approximately 8 weeks of age upon arrival served as subjects. Rats were housed in pairs in ventilated cages with enrichment and access to food as in Experiment 1. This experiment was run in two cohorts with 16 males and 16 females in the first cohort and 32 males in the second cohort. We used all males in the second cohort because no sex differences were observed in Experiment 1 and cannulae placement was more variable leading to more exclusions in the female rats in the first cohort. In combination with Experiment 1, these four cohorts served to replicate the diet effects four times.

#### Obesogenic diet

Rats were assigned to control and SCM groups as in the previous experiment in a counterbalanced manner according to starting weight. SCM was continuously available for 5 weeks prior to surgery. Following 24 h of recovery postsurgery, rats were given SCM for at least one additional week as the surgeries were performed over multiple days. In total, all animals had 6 weeks of SCM or chow access prior to training. The obesogenic diet was terminated prior to operant training when all animals were placed on a restricted chow diet.

#### Surgery

Stereotaxic surgeries were performed under aseptic conditions (maintaining sterile tip methods at a minimum). Rats were anaesthetized with isoflurane (4% induction; 2–3% maintenance). Metacam (2 mg/kg) and bupivacaine (1 mg/kg) were administered prior to surgery for analgesia. 1.0 ml of Ringers solution was administered to improve hydration and aid in recovery. A digital stereotaxic instrument (Kopf) was used to take coordinates at bregma and a drill (Foredom) was used to make two holes in the skull above DMS coordinates. The guide holes and bilateral cannulae (P1 Technologies (formerly PlasticsOne), 26 gauge) were implanted into the dorsomedial striatum of rats (AP: + 1.0 mm, ML: + /- 1.5, DV:−2.8 mm; 33-gauge internal cannulae extended 2 mm below guide cannulae). At least three screws on at least two different plates in the skull were screwed partway into the skull to create a surface for cement. The screws and cannulae were then secured in place with dental cement (Keystone Industries) to make a headcap. Dummy cannulae were inserted into the guide cannulae and stainless-steel caps were screwed down over them. Additional Metacam (2 mg/kg) was administered 24 and 48 h postoperatively. Rats were given at least one week (and not more than 10 days) to recover from surgery during which time those in the SCM group continued to receive SCM prior to food deprivation.

#### Training

Training proceeded in the same manner as with the second cohort in Experiment 1 apart from chocolate pellets (TestDiet LabTab sucrose chocolate 45 mg pellets) being utilized in place of liquid sucrose as a reinforcer for half of the rats.

#### Infusions

At least one mock infusion was performed on rats prior to the first infusion day to minimize effects of mechanical manipulation and restraint stress on performance. In these mock infusions, dummy cannulae were removed and internal cannulae were inserted. On test days, internal cannulae were inserted and 0.5 µl of solution (sterile saline or 1 μg LY379268 per 0.5 μl sterile saline) was bilaterally infused across 2 min. The dose used was based on the existing literature [27, 33, 35]. Internal cannulae were left in place for one minute following the end of the infusion to allow for drug diffusion. Dummy cannulae were then replaced and rats were returned to their cages. Tests occurred 5–20 min after infusion based on timing in the literature which ranged from 5–10 min [34] to 30 min [27]. Rats who showed any motor symptoms were not tested and were retested on a separate day with the same drug and prefeeding assignments.

#### Outcome-devaluation tests

Testing proceeded as in Experiment 1 except for infusions rather than injections occurring on test days. Rats received four infusions (two of LY379268 and two of saline), undergoing all four test conditions as in Experiment 1. Half of the rats in each diet condition received the same drug condition for tests one and two and were tested under both nondevalued and devalued conditions, then received the other drug condition for tests three and four under both devalued and nondevalued conditions.

#### Consumption test

As in Experiment 1, rats from one cohort were given a follow-up test where they received 1-hour free access to one of the two foods in the same manner as the devaluation tests. After 1 h the food was removed and fresh containers of each of the foods were placed into the cage for 10 min and consumption of each recorded. This was to confirm the effectiveness of specific satiety.

## Results

### Experiment 1

#### Weekly weight gain across the diet phase

As illustrated in Fig. 2A, all animals gained weight across weeks and the amount of gain was greater for those groups receiving SCM compared to those receiving only chow. This is supported by main effects of diet [*F*(1,52) = 14.074, *p* < 0.001], week [*F*(5,260) = 1394.3, *p* < 0.001] and a diet x week interaction [*F*(5, 260) = 33.973, *p* < 0.001]. There was a main effect of sex [*F*1,52) = 373.116, *p* < 0.001] and a sex × week interaction [*F*(5,260) = 147.943, *p* < 0.001] indicating that males, in addition to weighing more than females overall, also gained more weight across weeks. There was no interaction between sex and diet nor between sex, diet and week [*F*s<1].

#### Instrumental training

As shown in Fig. 2B, all animals acquired the instrumental response, increasing responding across days [*F*(6, 312) = 131.901, *p* < 0.001]. Animals in the SCM groups responded numerically less overall [*F*(1,52) = 3.89, *p* = 0.054] and their increase in responding tended to be less across days [day x group interaction; *F*(6,312) = 2.022, *p* = 0.062] though neither of these effects reached significance. There was no effect of sex [*F*(1,52) = 1.54, *p* = 0.22] and no interactions, involving sex [largest *F* = 1.3].

#### Outcome-devaluation tests

##### Early te﻿s﻿t

As shown in Fig. 2C all groups showed sensitivity to outcome devaluation [*F*(1,24) = 8.6, *p* = 0.007]. There was no effect of diet, sex or interactions with these factors in the early test [all *F*s <1].

##### Effects of LY379268 on sensitivity to outcome devaluation

Across the four tests, six rats did not meet the con3sumption criterion in one of the tests. They were not tested that day but retested the following day under the same condition and those data added to the final data set. There was a main effect of the cohort [data not shown; *F*(1,47) = 51.648, *p* < 0.001] driven by the fact that rats from cohort 1 responded less than those from cohort 2, perhaps due to previous extinction tests and/or shorter training sessions. However, the cohort did not interact with devaluation, diet, or sex, and so we combined data from the two cohorts in subsequent analyses. There was no main effect of sex [*F*(1,47) = 0.227, *p* = 0.635] and no interactions involving sex and so we collapsed across the factor of sex for further analyses. Preliminary analyses included order as a factor (i.e., whether LY379268 vs. saline was given in the first or second pair of tests). There was no main effect of order [*F*(1,47) = 0.024, *p* = 0.877] and no interactions involving this factor and so we collapsed across this variable in subsequent analyses.

There was a main effect of devaluation [*F*(1,53) = 48.806, *p* < 0.001] and while there was no main effect of diet [*F*(1,53) = 0.839, *p* = 0.364], there was a significant interaction between devaluation and diet [*F*(1,53) = 9.331, *p* = 0.004], replicating previous results. There was a main effect of drug [*F*(1,47) = 5.688, *p* = 0.021] with rats responding less overall following administration of LY379268. Drug did not interact with diet or devaluation [*F*s <1], but there was a marginal three-way interaction between drug, diet and devaluation [F1, 47) = 3.590, *p* = 0.062]. To investigate this further, and to test our hypothesis that LY379268 would rescue a diet-induced impairment, we examined the effect of LY379268 on sensitivity to devaluation in each the Chow and SCM groups. As shown in Fig. 2E, in the Chow group, there was a robust effect of devaluation [*F*(1,27) = 31.16, *p* < 0.001] but no drug x devaluation interaction [*F*(1,27) = 0.415, *p* = 0.525] as the drug did not improve the already clear devaluation effect. There was no main effect of drug [*F*(1,27) = 2.874, *p* = 0.102]. As shown in Fig. 2F, in the SCM group, there was no main effect of drug [*F*(1,26) = 2.24, *p* = 0.147], but a significant effect of devaluation [*F*(1,26) = 19.101, *p* < 0.001] and importantly, treatment with LY379268 improved sensitivity to devaluation evidenced by a drug x devaluation interaction [*F*(1,26) = 8.453, *p* = 0.007]. Pairwise comparisons further confirmed a significant devaluation effect in SCM animals after LY379268 [*t*(26) = 5.958, *p* < 0.01] but not after saline treatment [*t*(26) = 1.853, *p* = 0.080]. These results cannot be accounted for by differences in consumption during testing as similar amounts of food were consumed by rats in the SCM and Chow groups [*F*(1,53) = 2.9, *p* = 0.094]. Mean grams (SEM) consumed for Chow rats were 12.9 (1.2), 13.9 (1.2), 12.9 (1.3), 12.7 (1.3) in the LY-devalued, LY-nondevalued, saline-devalued, and saline-nondevalued, conditions, respectively. Mean grams (SEM) consumed for SCM rats were 12.1 (1.3), 12.8 (1.0), 13.5 (1.2) and 11.9 (1.2) for the LY-devalued, LY-nondevalued, saline-devalued, and saline-nondevalued, conditions, respectively.

#### Consumption test

As shown in Fig. 2D, a consumption test conducted on a separate day verified that devaluation by outcome-specific satiety was effective and not different between diet groups. Again, there was no difference in the amount consumed by the two groups in the initial prefeeding [*F*(1,52) = 0.616, *p* = 0.439]. Mean (SEM) consumption in grams was 14.0 (1.7) and 13.2 (1.6) for rats in the Chow and SCM groups, respectively. ANOVA for the consumption test that followed prefeeding revealed an effect of devaluation [*F*(1,52) = 43.675, *p* < 0.001], but no effect of diet [*F*(1,52) = 0.01, *p* = 0.919] or sex [*F*(1,52) = 2.832, *p* = 0.098], and no interactions involving these factors [all Fs <1].

### Experiment 2

**Fig. 3:**
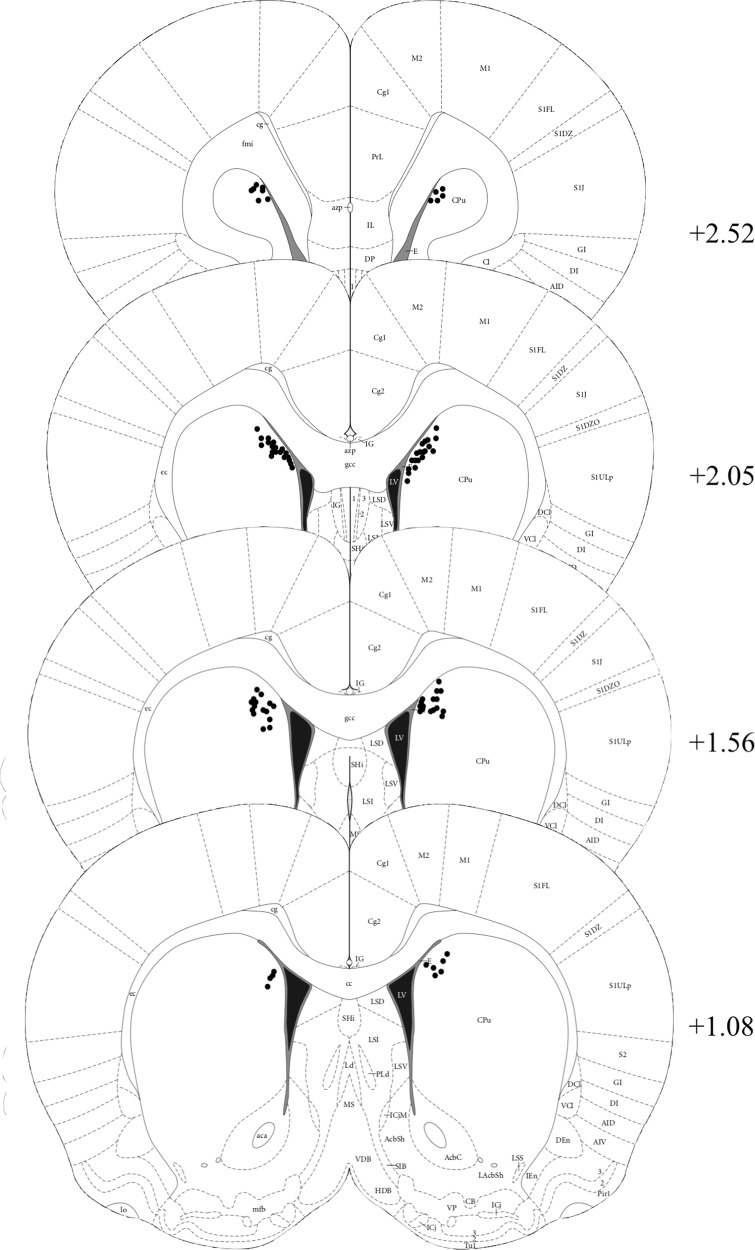
Schematic representation of cannula placements in the DMS for animals included in the behavioural analyses. Templates adapted from Paxinos & Watson, 2005. Numbers indicate distance from Bregma (mm) in the anterior-posterior plane. N = 29 for the Chow and 24 for the SCM groups, respectively.

Of the 64 rats, one female rat (in the SCM group) had to be euthanized because she failed to recover fully from surgery. Ten rats were removed from analysis due to misplaced cannula (See Fig. 3 for cannulae placements of animals included in the behavioural analyses [39]). Misplaced cannulae were not analyzed separately because most misplacements included unilateral placement in the DMS and unilateral placement in a ventricle meaning drug spread would be difficult to estimate. As such, 53 rats were included in the behavioural analyses with 11 females (7 Chow and 4 SCM) and 42 males (22 Chow and 20 SCM). The experimenter was blinded to the experimental group of the animals during histology analysis.

#### Weekly weight gain across the diet phase

**Fig. 4:**
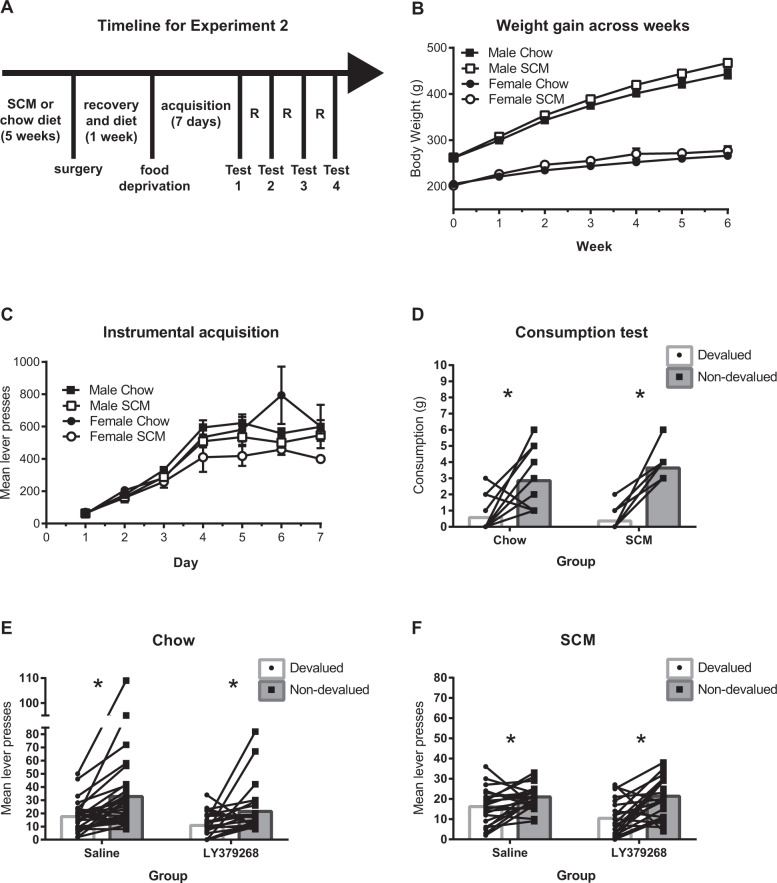
mGluR2/3 agonism specifically within the DMS is sufficient for restoring goal-directed control following long-term access to an obesogenic diet. A Experimental timeline for Experiment 2. **B** Weight gain. All rats gained weight across six weeks of SCM or Chow diets, while those in the SCM groups gained more than those receiving only chow and males gained more than females. Mean (SEM) body weight across weeks of diet exposure for male and female rats. **C** Instrumental acquisition. Mean (SEM) lever presses across days of instrumental training. Lever-press rates increased across days of training for all groups. **D** Consumption test of outcome devaluation. Chow and SCM groups both showed sensitivity to outcome devaluation as measured by consumption of a devalued versus nondevalued food verifying the efficacy of the devaluation treatment itself in both groups. **E** Outcome-devaluation test: Chow group. Rats maintained on chow are sensitive to outcome devaluation and treatment with LY379268 reduces overall responding. **F** Outcome-devaluation test: SCM group. A history of access to an obesogenic diet reduces sensitivity to outcome devaluation. This deficit was ameliorated by mGluR2/3 agonism in the DMS. N = 29 for the Chow and 24 for the SCM groups, respectively. Across all figures, * indicate that p < .05.

Rats gained weight across the six-week diet period [*F*(6300) = 616.38, *p* < .001]. There was also a significant interaction between week and diet [*F*(6, 300) = 3.30, *p* = .004] and week and sex [*F*(6, 300) = 145.01, *p* < .001]. There was a main effect of sex on weight [*F*(1, 50) = 235.51, *p* < .001] but no main effect of diet [*F*(1,50) = 1.98, *p* = 0.165]. There was no week by sex by diet interaction [*F*(6,300) = 0.45, *p* = 0.843]. Together, these results show that rats with access to SCM gained more weight than the group with just Chow access and males gained more weight than females (See Fig. 4A for a timeline of Experiment 2 and Fig. 4B for weight gain across diet).

#### Instrumental training

All rats learned to lever press across training as shown in Fig. 4C. A mixed measures ANOVA yielded main effects of day [*F*(6, 282) = 100.75, *p* < .001] and of diet [*F*(1, 47 = 5.23, *p* = .027] indicating that overall responding increased across days and that the SCM groups responded less than chow groups. There were also interactions between diet and day [*F*(6, 282) = 3.34, *p* = .003], sex and day [*F*(6, 282) = 3.03, *p* = .007], and a marginal three-way interaction [*F*(6, 282) = 2.05, *p* = .059]. However, these interactions seem to be primarily driven by variability on day 6 of training. Analyses omitting day 6 show a main effect of day [*F*(5, 235) = 105.97, *p* < .001] and a marginal effect of diet [*F*(1,47) = 3.99, *p* = 0.051] but no interactions. Finally, a one-way ANOVA on the last day of training shows no differences in lever-pressing between groups [*F*(3,49) = 1.17, *p* = .329]. These analyses and results from Experiment 1 suggest that overall, there is not a consistent sex difference or effect of diet on instrumental responding by the end of acquisition.

#### Outcome-devaluation tests

Across the four tests, seven rats did not meet minimum consumption criteria during satiation. They were not tested but retested under the same conditions the following day. Multiple rats (*N* = 16: SCM = 10, Control: 6) appeared lethargic immediately following the first infusion. We were hesitant to alter our dose due to the findings of Myal et al. [33] and Cannady et al. [40] who showed no behavioural effects with a lower intracranial dose of LY379268 and because the 1ug/.5ul dose has been frequently reported for intracranial infusions [27, 33, 35]. This dose was smaller than that infused by Bossert et al. (3ug/.5ul) [34] into the nucleus accumbens though they reported no motor effects. Cartmell et al. [41] showed that high doses (10, 30, and 100 mg/kg) of LY379268 given orally impaired motor function as measured by rotarod performance (they saw no motor impairments with doses up to 3 mg/kg). Interestingly, there were no motor impairments following 4 days treatment with even the high doses of LY379268, indicating some sort of tolerance to motor effects. Similarly, in our hands, these effects were transient, occurring predominantly on the first day of LY379268 infusion. Animals that showed signs of motor impairments were not tested but returned to the home cage and retested under the same conditions the following day. Motor impairments were generally not observed in these subsequent tests.

Preliminary analyses showed no sex differences or drug order effects in test performance and so we collapsed across these variables in our analyses. ANOVA revealed a main effect of drug [*F*(1, 41) = 7.97, *p* = .007] with animals responding less overall after LY379268 and a main effect of devaluation [*F*(1,41) = 24.69, *p* < .001] due to less overall responding in the devalued condition. There was no main effect of diet [*F*(1, 41) = 2.15, *p* = 0.150], however, importantly, there was a three-way interaction between diet, drug, and devaluation [*F*(1, 41) = 5.82, *p* = .020]. To explore this interaction, we ran separate 2 ×2 ANOVAs examining effects of drug and devaluation for SCM and Chow groups. As shown in Fig. 4E, in the Chow condition, as expected, there was a main effect of devaluation [*F*(1,22) = 15.678, *p* < .001], and also a main effect of drug [*F*(1,22) = 7.21, *p* = .014]. Importantly, there was no drug x devaluation interaction [*F*(1, 22) = 1.12, *p* = .302], indicating that the reduced responding seen from LY379268 equally affected both devalued and nondevalued tests. When we examined the SCM group, we saw a different pattern of effects. As shown in Fig. 4F, there was a main effect of devaluation [*F*(1,19) = 10.85, *p* = .004] but no main effect of drug [*F*(1,19) = 1.53, *p* = .231. Crucially, there was a significant drug by devaluation interaction [*F*(1,19) = 8.48, *p* = .009]. Pairwise comparisons revealed that for the rats who had received the obesogenic diet, there was a significant difference between nondevalued and devalued responding for both vehicle [*t*(22) = 2.28, *p* = .033 and LY379268 treatment [*t*(20) = 3.55, *p* = .002], though as suggested by Fig. 4F, and supported by the significant interaction, sensitivity to devaluation improved following treatment with LY379268. There was no effect of diet or interaction of diet with devaluation on consumption in the hour-long satiety period (Largest *F* < 1). Mean grams (SEM) consumed during satiation for Chow rats were 6.89 (0.55), 7.79 (0.67), 7.18 (0.53), and 7.54 (0.52) in the LY-devalued, LY-nondevalued, Veh-devalued, and Veh-nondevalued groups, respectively. For SCM rats, the mean amounts consumed in grams (SEM) were 7.45 (0.62), 7.63 (0.58), 7.91 (0.57), and 7.09 (0.66) for LY-devalued, LY-nondevalued, Veh-devalued, Veh-nondevalued, respectively. This confirms no difference in consumption during satiety prior to the test. Overall, these results indicate that following long-term access to a high calorie, palatable diet, rats show less selective sensitivity to outcome devaluation, but that sensitivity to this treatment is improved by intra-DMS treatment with an mGluR2/3 agonist.

#### Consumption tests

An additional consumption test was performed on one cohort to confirm the efficacy of outcome-specific satiety. A mixed ANOVA yielded a main effect of devaluation [*F*(1, 21) = 90.98, *p* < .001]. There were no interactions between devaluation and diet ([*F*(1, 21) = 1.68, *p* = .21]; all other *F*s<1) though there was a trend toward a main effect of sex [*F*(1, 21) = 3.01, *p* = .097] with females tending to eat less overall (See Fig. 4D). This test suggests that diet-induced deficits in sensitivity to devaluation seen in the lever tests were not due to a reduced efficacy of the devaluation treatment itself.

## Discussion

We show here that long-term access to an obesogenic diet results in a disruption of goal-directed responding in both male and female rats. This deficit can be ameliorated by agonism of mGluR2/3 receptors, both systemically and specifically within the DMS. This adds to a growing literature that suggests that diet, like drugs of abuse, alters dorsal striatum function, which in turn alters behaviour by reducing goal-directed control.

A large literature has shown that parallel and interacting circuits control goal-directed and habitual behaviour: the dorsomedial striatum (DMS) mediates goal-directed behavior while the dorsolateral striatum (DLS) mediates habitual responding [37, 42–44]. However, it is unknown exactly how these two regions interact to promote and inhibit goal-directed behavior across normal circumstances, or how this regulation is changed in conditions where habitual responding occurs prematurely. Changes to synaptic proteins and activity following drug exposure point to several mechanisms that could underlie the changes to behavioural control. Furlong et al. [13] has shown that methamphetamine administration results in bidirectional changes in glutamatergic proteins in the DMS and DLS, with the DMS showing a reduction in density of glutamatergic receptor subunits and vesicular transporter proteins and the DLS showing an increase. The same methamphetamine exposure was shown to promote habitual behavioural control in a separate group of animals. This implies that methamphetamine may cause behavioural changes by simultaneously impairing goal-directed control in the DMS and amplifying habitual control via changes in the DLS, though this could also indicate compensatory control by a habit region. Related results by Jedynak et al. [45] show that pretreatment with methamphetamine results in an increase in mushroom and thin dendritic spines in the DLS and a decrease of these spines in the DMS. This implies that drugs of abuse may produce opposing effects on glutamatergic signaling and plasticity within the DMS and DLS. Finally, Corbit, Chieng, and Balliene [12] found that experimenter-administered cocaine given prior to training rats to lever press for food results in habitual responding. The same drug treatment leads to increased frequency of spontaneous and miniature excitatory postsynaptic potentials in the DMS but not the DLS, indicating that increased spontaneous glutamatergic activity within the DMS may be the cause of an impairment in goal-directed control. Behavioural deficits, as well as electrophysiological changes were prevented by administration of NAC, further implicating a glutamatergic mechanism. The data presented here show that goal-directed responding was improved by manipulating glutamate transmission within the DMS, the region thought to be essential for response-outcome encoding that underlies goal-directed performance [37]. Thus, one more basic implication of these findings is that obesogenic diet disrupts the ability of rats to respond in a goal-directed manner, and that the insensitivity to reinforcer devaluation may be due to an impairment in goal-directed control caused by SCM rather than a facilitation of habitual control via plasticity in habit regions. Nonetheless, dysfunction in DMS may result in earlier control by the DLS habit system which remains relatively intact. This accounts for why once habitual responding is established, manipulation of DLS, though not the source of pathology, still impacts behavioural expression [4]. Though we did not directly test if habit regions are affected, our data demonstrate that mGluR2/3 agonism within the DMS is sufficient to restore behavioural control in rats who previously received an obesogenic diet.

Restored goal-directed responding following administration of an mGluR2/3 agonist which should serve to dampen glutamate release [46], implies that obesogenic diet dysregulates glutamate and this results in responding that is insensitive to reinforcer devaluation. This is particularly interesting because lesion or inactivation of the DMS also results in impairments in goal-directed responding [37, 47], meaning that dysfunction, much like inactivation can disrupt sensitivity to devaluation. Disruption of outcome devaluation due to perturbation of neurons has been shown before in the orbitofrontal cortex in response to chronic ethanol exposure [18] and this mimics the insensitivity to reinforcer devaluation seen with orbitofrontal cortex lesions [48, 49], though this appears to be due to an increase in neural activity in response to lever-pressing and a decrease in activity corresponding to outcome delivery [18]. One explanation for how dysregulation of glutamate might function similarly to a lesion is through changes in feedforward inhibition onto cells important for maintaining outcome sensitivity. For example, glutamatergic activation of an interneuron might effectively cause more inhibition on striatal output neurons. Various striatal interneurons that directly inhibit MSNs also receive differing and direct input from cortical and/or thalamic glutamatergic afferents, and this is dependent on the interneuron subtype (for an excellent review see Tepper et al. [50]). Alternatively, some striatal cell types may be more sensitive to drug-induced excitotoxicity, for instance, methamphetamine administration reduces the number of striatal enkephalin expressing cells, parvalbumin-expressing interneurons, and cholinergic interneurons, without affecting numbers of somatostatin-expressing interneurons [51–53]. Therefore, a diet-induced disruption to the circuit might mechanistically function as a lesion based on the microcircuitry that is affected. However, our ability to rescue behaviour with mGluR2/3 agonism indicates that diet does not disrupt the circuit irreconcilably as a lesion would. Of interest, in a rat model of hypoxia-ischemia, mGluR2/3 agonism has been shown to reduce oxidative stress and reactive oxygen species production thus reducing brain injury [54]. Further, inhibiton of mGluR2/3 receptors blocks the ability of NAC to reduce neuroinflammation following chronic alcohol consumption [55] (also shown elsewhere to promote habitual control [36]) and so mGluR2/3 appears to have several neuroprotective effects that could contribute to the current findings.

The source of glutamate to the DMS that may be driving these effects is unknown but could arrive to the DMS via its many glutamatergic afferents from several cortical and thalamic regions. The most likely contributors are other regions known to be involved in goal-directed control, since our results show a deficit in DMS-mediated goal-directed control. The prelimbic cortex has been implicated in goal-directed learning and is heavily connected with the DMS [47–51]. Inhibition of the prelimbic cortex to DMS pathway (a pathway known to be glutamatergic) results in insensitivity to reinforcer devaluation [56, 57], and reduces operant responding at test generally [57]. This is similar to how the Chow rats in our experiment behaved with intra-DMS LY379268 (which would reduce glutamate release), showing a general reduction in responding. Further, prelimbic projections to the nucleus accumbens core have also been implicated in drug-induced reinstatement [58] and disconnection of the prelimbic-mediodorsal nucleus of the thalamus impairs goal-directed control [59]. Since these known connections of the prelimbic cortex have been implicated in similar behaviours, it is a reasonable future region to investigate as a source of glutamate to the DMS that is disrupted by obesogenic diet. The orbitofrontal cortex has also been implicated in outcome valuation and goal-directed control and its neurons are more active when behaviour is goal-directed and thus may be another important cortical input contributing to DMS activation here [49]. Additionally, thalamic input alone could result in the impairment in goal-directed control. Lesion of the mediodorsal thalamus impairs goal-directed responding [59, 60] and the mediodorsal thalamus is heavily interconnected with the DMS [61]. Finally, one study has shown that optogenetic stimulation of the anterior parafascicular nucleus of the thalamus glutamatergic terminals in the DMS is reinforcing, as mice will lever-press for stimulation[62]. This effect is mitigated by systemic injection of LY37926859. However, Bradfield et al. [63] show that Pf-DMS pathway inhibition impairs behavioural adaptations to contingency changes but not sensitivity to outcome value, indicating that it is not likely to be parafascicular thalamic inputs that are causing our effects. Overall, future work will determine the afferent projections to the striatum that are responsible for diet-induced changes.

In the current study, we gave continuous access to SCM and did not also examine restricted access as in our previous work [4] to model diet-induced obesity. While Furlong et al. [4] focused on the effects of restricted diet access, the behaviour of rats with continuous access to SCM in that study also differed from controls. In the current study, as expected, rats in the SCM groups gained more weight than those maintained on chow verifying the validity of this paradigm as a model of obesity. While not the focus of the current study, future experiments should investigate other potential physiological effects of this diet. Based on what is known about the effects of related diet manipulations, we might expect increased abdominal fat, insulin resistance, glucose tolerance, elevated leptin, and a decrease in gut bacterial diversity [64–66]. Although these characteristics of metabolic syndrome are often measured after 12 or more weeks of diet access, some changes are detected at six weeks and may be relevant to the current findings. Although rats receiving restricted access to SCM do not reliably gain weight compared to Chow controls, they do show impairments in goal-directed control and so it is also possible that the impairments here may not be directly related to weight gain.

The current study included both male and female subjects. While not surprising, females gained less weight than males. Importantly, however, we failed to detect any sex differences in the development of habits in Chow groups, in those receiving SCM, or in response to systemic mGluR2/3 agonism. These results are in apparent contrast to those of Schoenberg et al., [38] who found that female rats demonstrated insensitivity to outcome devaluation after relatively brief training where subjects earned only 160 outcomes before testing. Male rats showed a robust devaluation effect after the same amount of training. The reason for this difference is unknown. While we explored the possibility that we too would see deficits after brief training in Experiment 1, because we were primarily interested in the effects of diet, we did not pursue this matter further. The possible contribution of several procedural differences including the method of devaluation (conditioned taste aversion vs. outcome-specific satiety), the instrumental response (nose-poke vs. lever-press) and the final reinforcement schedule (VI30 vs. VI60) could be explored in future research.

These results may help elucidate treatments for restoring behavioural control in those who have had chronic exposure to obesogenic diets and who struggle to alter food-related habits. Although mGluR2/3 agonists are not approved for use in humans, a related mechanism can be targeted with NAC, which is available over the counter, is well-tolerated, and has shown efficacy in clinical trials treating a variety of substance use and psychiatric disorders [64, 65]. It also has performed well in clinical trials aimed at improving complications arising from obesity (for a thorough recent review see Dludla et al. [67]). Further, in a rat model of diet-induced obesity, NAC reduces binging on high fat/high sugar pellets, elevation of lever-pressing across training for high fat/high sugar pellets and responding during a stimulus that signals food unavailability [68]. We show here that mGluR2/3 agonism, one indirect mechanism of action of NAC, can ameliorate obesogenic-diet-induced deficits in goal-directed control. This could indicate that NAC could help treat not only complications from obesity but might improve goal-directed control to reduce habits associated with the continued eating of foods that contribute to overweight and obesity. This may mark a two-pronged drug approach to a clinical issue where behaviour is a critical contributor to continued dysfunction.

These results also suggest that mGluR2/3 agonism may restore impairments in goal-directed control following exposure to drugs of abuse or other insults. Corbit, Chieng, and Balleine [12] showed that NAC could normalize miniature EPSPs in the DMS and goal-directed control in rats who had received a pretreatment with cocaine. Since the mechanism of NAC is partially overlapping mGluR2/3 agonism, and NAC showed efficacy in the same paradigm with a different insult, behavioural deficits following exposure to other drugs of abuse may also be ameliorated by mGluR2/3 agonism. Further studies should examine this.

While long-term exposure to SCM might affect rats’ subsequent levels of hunger or reactions to other palatable foods, a difference in consumption during the induction of outcome-specific satiety does not explain our results. Analysis of separate consumption tests where rats have free access to one food for an hour to devalue it as in the other tests and are then presented with both foods and consumption of each measured, show that regardless of diet history, rats prefer the nondevalued food, thus devaluation is intact. Similarly, consumption during satiation across the primary devaluation tests did not differ by diet. Finally, while animals in the SCM groups responded numerically less during training, their lack of sensitivity to devaluation cannot be explained by a floor effect since the improved sensitivity to devaluation following LY379268 treatment was driven by a further reduction in responding in the devalued condition.

Overall, these results show that dysregulation of DMS glutamate is a mechanism through which an obesogenic diet may reduce goal-directed control. Though this is not a comprehensive analysis of all the brain regions that are dysregulated by diet, we show that mGluR2/3 agonism within the DMS is sufficient to restore behavioural control. This contributes to the current drug literature that has shown that LY379268 administration reduces drug self-administration and reinstatement by adding that it additionally can restore goal-directed control. Regulation of glutamate signaling provides a promising treatment approach to promote behavioural flexibility which can hopefully help to prevent cycles of overeating which serves to dysregulate glutamate and, in turn, further impairs goal-directed control.
